# Toothache

**Published:** 1883-07

**Authors:** 


### TOOTHACHE.

Dr. Guillott claims to have had very good results in the treatment of toothache from the injection of chloroform beneath the mucous membrane of the gum. The effects are more immediate and lasting than those of morphine. There have been no resultant abscesses or inflammation.—*Progres Medical.*

This is a fair specimen of the paragraphs regarding the mouth and teeth which one often finds in foreign medical journals. Supposed-to-be-learned-Professors, with half the alphabet strung to their names like the knotted tail of a kite, will, with owl-like gravity, lofty condescension, and the most priggish air conceivable, inform an anxiously listening world that *aqua camphoræ* is excellent for toothache. It would be quite as definite to say that *sulphate of quinia* is good for illness.

To the dentist the term "toothache" is devoid of anything but a general meaning. There are so many kinds of toothache, and the pains in teeth arise from so many causes, some of which are not at all connected with these organs, that it would seem as if learned writers should not exhibit their ignorance in the manner in which many of them do. Chloroform injected beneath the gum may temporarily relieve an irritable nerve filament without subsequent abscess, but how would it affect a violent case of periosteal inflammation? Tincture of aconite would possibly relieve an inflamed tooth pulp, but of what benefit would it be in a case of exostosis? Toothache, like other pains, is but the symptom of a diseased condition, and all the nostrums sold by quack medicine venders, or prescribed by physicians who know nothing of the pathology of the case in hand, cannot work a cure. There are few intelligent dentists who will not cite numerous instances in which a suffering patient has been under the care of a physician, for weeks perhaps, until in despair he was turned over to the dentist, who wrought a cure in a moment. We have known people who were frightened out of their wits because some physician had long treated an impacted tooth as an osseous tumor. We were once summoned to attend an operation for necrosis, but which a very slight examination enabled us to diagnose as alveolar abscess, with induration. There are no tissues or organs of the body which physicians study so little as the teeth and their relations, and yet, plentiful as are competent dentists, there are too many who are continually recommending worthless nostrums, rather than to turn them over to the qualified specialist.

